# Neurological Manifestations in Pediatric Patients Hospitalized for COVID-19: Experiences of the National Medical Center “20 de Noviembre” in Mexico City

**DOI:** 10.3390/children9050746

**Published:** 2022-05-19

**Authors:** Brian Javier López-Pérez, Diana Alejandra Cruz-Chávez, Elsa Solórzano-Gómez, José Antonio Venta-Sobero, Iván Alejandro Tapia-García, Christian Gabriel Toledo-Lozano, Andrea Torres-Vallejo, Gabriela Vianney Castro-Loza, Yazmín Evelyn Flores-Jurado, Cristal Lucero Hernández-Soriano, Sofía Lizeth Alcaraz-Estrada, Paul Mondragón-Terán, Juan Antonio Suárez-Cuenca, Silvia Garcia

**Affiliations:** 1 Department of Pediatric Neurology, Centro Médico Nacional “20 de Noviembre”, ISSSTE, Mexico City 03229, Mexico; bjlp91@gmail.com (B.J.L.-P.); dacch_taz@hotmail.com (D.A.C.-C.); solorzanoeg@gmail.com (E.S.-G.); ventadoc@yahoo.com.mx (J.A.V.-S.); even.fj@gmail.com (Y.E.F.-J.); crissspedia@gmail.com (C.L.H.-S.); 2 Department of Pediatric Infectology, Centro Médico Nacional “20 de Noviembre”, ISSSTE, Mexico City 03229, Mexico; ivanalejandrotapiagarcia@gmail.com; 3 Department of Clinical Research, Centro Médico Nacional “20 de Noviembre”, ISSSTE, Mexico City 03229, Mexico; drchristiantoledo@gmail.com (C.G.T.-L.); p.mondragonteran@gmail.com (P.M.-T.); juanantonios55@gmail.com (J.A.S.-C.); 4 Department of Pediatric Endocrinology, Instituto Nacional de Pediatría, Mexico City 03700, Mexico; andreatovall@gmail.com; 5 Department of Urdergraduate Research, Hospital Militar de Especialidades de la Mujer y Neonatología, Mexico City 11200, Mexico; freezepop11@hotmail.com; 6 Department of Genomic Medicine, Centro Médico Nacional “20 de Noviembre”, ISSSTE, Mexico City 03229, Mexico; sofializeth@gmail.com; 7 Department of Neuroscience, Centro Médico Nacional “20 de Noviembre”, ISSSTE, Mexico City 03229, Mexico

**Keywords:** COVID-19, SARS-CoV-2, neurological manifestations, children

## Abstract

COVID-19 has affected millions of children and, while it was previously considered as a respiratory disease, neurologic involvement has also been documented. The objective of this study was to identify the neurological manifestations (NMs) and the outcomes of children with COVID-19 who attended the National Medical Center “20 de Noviembre”. Methods: A retrospective cohort study of children hospitalized for COVID-19 from April 2020 to March 2021 was conducted. Clinical-demographic data were registered. Neurologic manifestations were defined as any clinical neurological expression of the central and/or peripheral nervous system that occurred during admission or hospitalization. Results: In total, 46 children with a confirmed COVID-19 result, 26 (56.5%) boys and 20 (43.5%) girls with a median age of 8.9 ± 4.6 years, constituted the study population. Half of the children showed some NMs, and this group of patients concomitantly showed acute lymphoblastic leukemia (ALL, 56%), obesity (17.3%), or acute myeloblastic leukemia (AML, 4.3%). The most frequently described NMs were headache (13, 56%), encephalopathy (10, 43.47%), and epilepsy (4, 17.39%). The mortality rate in children with NMs was 21.7% and they had a higher mortality rate when compared to those without NM *p* ≤ 0.025. Conclusions: NMs occurred predominantly in male children aged 6 to 12 years; ALL was the most frequent comorbidity. Headache prevailed and hypoxemia, hypocalcemia, elevated ferritin, and C-reactive protein were associated with NM. Finally, NMs were a risk factor for mortality.

## 1. Introduction

The current global pandemic of COVID-19 caused by the novel human coronavirus SARS-CoV-2 has posed enormous and unique challenges for entire medical systems and health care professionals. Neurological involvement is increasingly recognized among adults with COVID-19, with the most common symptoms including headache and anosmia or ageusia, while the most common neurological signs and/or syndromes are acute encephalopathy, coma, and stroke [1]. Neurological manifestations (NMs) associated with COVID-19 have been relatively rare in neonates and children. In children, headache and acute encephalopathy are most frequently described. Children with neurological manifestations were more likely to require an intensive care unit [2]. However, reports suggesting neurological dysfunction in this age range are increasing [3].

Currently, children and adolescents under the age of 20 years represent 21% of the reported cases of COVID-19 [4]. These patients usually experience mild to moderate disease and primarily present with pulmonary symptoms [5]. Children with severe COVID-19 disease often have an underlying comorbidity, such as obesity, elevated inflammatory markers [6], or immunosuppression [7,8].

In COVID-19, neurological involvement is frequent, and patients tend to develop symptoms, such as sensory deficits in smell and taste, delirium, encephalopathy, headache, cerebrovascular accidents, and PNS disorders (myopathy and neuropathies) [3,9]. Early reports described a broad spectrum of neurological conditions associated with SARS-CoV-2 infection in adults, with symptoms including anosmia/ageusia, headache, dizziness/ataxia, psychosis, dementia, depression, anxiety, mania, acute encephalopathy, encephalitis, necrotizing encephalopathy, epilepsy, Guillain-Barre syndrome (GBS), posterior reversible encephalopathy syndrome, and acute ischemic or hemorrhagic stroke [9,10,11,12,13,14,15,16,17,18,19]. Rare neurological complications include intracranial hemorrhage, cranial nerve palsy, Guillain-Barre syndrome and vision problems [20].

Hospitalized pediatric patients with COVID-19 and multisystemic inflammatory syndrome may present with neurological involvement, for which, unlike adults, most symptoms are transient [21]; however, the mid-term and long-term course of these symptoms is uncertain.

In children, multi-systemic inflammatory syndrome (MIS-C) may be accompanied by neurological manifestations (NMs) involving the central nervous system (CNS) and/or peripheral nervous system (PNS) with different and unpredictable degrees of severity, including the risk of death [22,23].

In the case series published between March and August 2020, the frequency of neurological symptoms in children and adolescents with COVID-19 was highly variable, ranging from 6% to 58% of hospitalized patients with MIS-C with CNS and/or PNS involvement [23,24,25].

COVID-19 is less frequent in children and neurological manifestations are rare in Asian, European, and American populations [26]; however, the Latin American population is most likely underrepresented in the current literature base.

The aim of this study was to identify the NMs and outcomes of children with COVID-19 who were treated at the National Medical Center “20 de Noviembre”.

## 2. Materials and Methods

To characterize patients that showed NMs, a retrospective cohort study was carried out on patients hospitalized with COVID-19 at the Centro Médico Nacional “20 de Noviembre” ISSSTE in Mexico City from 1 April 2020 to 31 March 2021.

Data regarding age, gender, and comorbidities (obesity, asthma, hematology, and other diseases history) were collected from electronic medical records.

Typical presenting symptoms since COVID-19 onset (fever, cough, dyspnea, diarrhea, throat pain, rhinorrhea, arthralgia, myalgia, general discomfort, and abdominal pain) were provided by patients or their parents. Any missing or uncertain records were collected and clarified through direct communication with health care clinicians.

All patients with respiratory symptomatology or pulmonary disease were evaluated in a triage service. Enrolled patients met the following inclusion criteria at the hospital: pediatric inpatient, either male or female, ranging from 1 to 17 years, 11 months and 29 days (divided into groups of children under 3 years old, 3 to 5 years old, 6 to 12 years old, and 12 to <18 years old), with SARS-CoV-2 PCR-positive tests. The following data were obtained from clinical electronic records: age, gender, date of admission, history of diabetes mellitus, obesity, hematological diseases, asthma, or any another comorbidity (including pinealoblastoma, bronchopulmonary dysplasia, rhabdomyosarcoma, germinoma, teratoma, germ cell tumor, myelodysplastic syndrome, Hodgkin’s lymphoma, medulloblastoma, ectodermal dysplasia, osteosarcoma, diabetes mellitus 1, and systemic lupus erythematosus). Likewise, the presence of cough, odynophagia, rhinorrhea, dyspnea, myalgia, arthralgia, fever, chest pain, diarrhea and the date of onset of symptoms were registered. Due the limitations regarding access to imaging studies, with priority placed on chest CT, only those patients with suspicious stroke had a neuroimaging study performed. Any neurological manifestation from both the CNS and PNS that had occurred at admission or during hospitalization was considered a neurological symptom. Children were divided into two groups according on whether they showed neurological manifestations. Cases whose information was confusing were eliminated. The outcome of each child was documented according to their clinical condition at the time of discharge from the hospital as (1) asymptomatic, (2) respiratory symptoms, (3) neurological symptoms, (4) respiratory and neurological symptoms, or (5) death.

Statistical analysis. All demographic-clinical data and mortality during hospitalization are presented as n (%). Continuous variables are presented as mean and SD. For inferential analyses, the Chi square and OR CO 95% or *t*-test were used as appropriate. Statistical significance was considered if *p* ≤ 0.05. Data were analyzed using SPSS-23.

## 3. Results

In total, 52 inpatients met the selection criteria; 4 cases were eliminated due to incomplete data in the clinical record, and 2 cases were relocated to another hospital due to administrative issues.

In total, 46 inpatients with a confirmed SARS-CoV-2 infection diagnosis were recruited. The mean age was 8.9 ± 4.6 years, 26 patients (56.5%) were male, and most (43.5%) were school children. The study population was divided into an NM and non-NM group, which showed no significant differences regarding age or sex. The clinical-demographic data are shown in Table 1.

The most prevalent comorbidity was acute lymphoblastic leukemia. In the group with NM, the most frequent comorbidity was ALL (56.5%) while in non-NM, 43.5% presented with other comorbidities (Table 2).

Regarding symptoms, fever, cough, headache, dyspnea, diarrhea, encephalopathy (as evaluated by any diffuse disturbance of brain function, with acute onset) and general discomfort were most frequently reported, as described in Table 3.

Regarding organ function and metabolic parameters, the NM group showed higher values of ferritin, C-reactive protein, DHL, PO2, and Kirby quote and lower hemoglobin and calcium, as shown in Table 4.

From the total sample, 15 patients were admitted to the pediatric intensive care unit, of whom 14 required ventilatory support with assisted mechanical ventilation and 11 required vasopressor support. Steroid therapy was used in 30 cases, enoxaparin in 24 cases, and tocilizumab in 9 cases. One patient with chronic kidney disease received renal replacement therapy with hemodialysis.

The duration of hospitalization was 31.4 ± 33.3 days in the NM group and 28 ± 28.8 days in the non-NM group (*p* ≤ 0.91). When the endpoint was a combination of outcomes (asymptomatic discharge, discharge with respiratory symptoms, and death), the outcome was associated with NMs (Chi squared *p* = 0.043); however, NMs resulted in the prevention of asymptomatic discharge and risk of death (OR 0.1 and OR 2.2, respectively). Patients with NMs were discharged until remission.

Among patients that died (n = 5, 10.86%; 4 died due to acute respiratory failure and 1 patient developed intracranial hemorrhage), all showed NMs, haemato-oncological pathology, and encephalopathy, of which 2 patients presented with headache, 1 had seizures, and 1 experienced cerebral hemorrhage (Table 5).

## 4. Discussion

In this study, we analyzed COVID-19 manifestations in Mexican pediatric patients. The study population included 46 children aged 8.9 ± 4.6 years who were predominantly male (56.5%) and had a confirmed SARS-CoV-2 infection. Our demographic findings are similar to previously published work in different countries involving pediatric patients with COVID-19 [6,9,20,26]. One study reported a mean age that was similar to our findings [9] and another study also demonstrated a higher incidence in boys compared to girls [26].

In this study, we observed that half of the population experienced NMs, in contrast with that reported in other studies, which showed an NM incidence of 22% or less in children [6,9]. Similar to previous reports, boys had a higher incidence of NM than girls [6,9,26]. Mean age did not significantly influence the presence of NM, which is concordant with a previous study [6]. Headache was the most frequent neurological symptom, followed by encephalopathy and epileptic seizures, as previously reported [6,9].

Another finding was that in hospitalized children with moderate or severe COVID, all children presented with comorbidities. For patients with NM, the most prevalent comorbidities were haemato-oncologic diseases, ALL (56%), AML (4.3%), and obesity (17.3%). Other studies have reported obesity, asthma, and immunosuppression [15] or found that children with neurological symptoms were previously healthy [9]. 

Hypocalcemia has been linked with a worse prognosis in COVID-19, but no clear explanation is currently available [27]. We found decreased levels of calcium in children with COVID-19, and serum inflammatory biomarkers, increased ferritin, and decreased blood oxygen saturation and hemoglobin, which was associated with higher mortality [24]. The sub-group of children with NM was characterized by a lower oxygen saturation and serum calcium levels, and higher ferritin and C-reactive protein levels. In our study, 10.9% of the patients died, all of whom were neurologically compromised, which varies from previous similar studies [9,20].

Regarding NMs associated with COVID-19, similar to what was observed in this study, multiple NMs have been reported in adults: headache, encephalopathy, seizures, and status epilepticus, among others [3,9,28,29,30,31], of which non-specific headache is the more prevalent NM [32,33,34,35].

Regarding the laboratory parameters, analogous to the findings in pediatric patients with COVID-19, C-reactive protein is a marker that can predict the prognosis of COVID-19 [36]. In this case, elevated values have been associated with worse outcomes [37]. Elevated values of this biomarker were observed in this study, probably due to the presence of comorbidities in the patients included.

Our results showed that the most frequent comorbidities in this population of children with COVID-19 were hemato-oncological diseases and obesity. Similarly, in other studies, common comorbidities reported in patients with COVID-19 include obesity, hypertension, cardiovascular disease, diabetes, etc. [26,37,38,39]. In the Mexican population, patients reported at least one comorbid condition, mostly obesity, followed by diabetes and arterial hypertension, which have been identified as relevant risk factors regarding the acquisition and development of severe infection [40]. Furthermore, several investigations have shown that pre-existing conditions predispose patients with COVID-19 to an adverse clinical course and an increased risk of intubation and death [37], and higher case fatality rates compared to those without comorbidities [41].

Cancer is an important comorbidity associated with poor outcomes in patients with COVID-19, especially hematological and pulmonary, as they are more likely to develop complications [37,42]. However, it has been suggested that in most patients who die from COVID-19, such patients’ pre-existing medical conditions contribute to death rather than being the direct cause [39].

Our study has several limitations: the analyzed population was heterogeneous regarding its demographic characteristics as the patients had different sociocultural levels, all hospitalized patients were diagnosed with COVID-19, and no follow-up of the patients included in the study was conducted. The findings presented, however, contribute to the identification of NMs in children, among whom, although they represent a population in which the disease is less frequent and cases tend to be less severe compared to adults, it can be severe, and NMs are a risk factor for higher mortality. It is important to emphasize the lack of publications on pediatric patients with NMs and SARS-CoV-2 in the Latin American population. At the time of discharge, the patients did not present neurological symptoms; however, follow-up of the patients is required to observe symptoms in the short and long term.

## 5. Conclusions

In this study, neurological involvement was common in children hospitalized with COVID-19. NMs predominantly occurred in male school children. All children with NMs presented with comorbidities, among which hemato-oncological diseases were the most frequent, with ALL being the most frequent comorbidity.

Headache, encephalopathy, and epileptic seizures were the most frequent NMs. Hypoxemia, hypocalcemia, elevated ferritin, and C-reactive protein were associated with NMs. Finally, NM was a risk factor of mortality.

Patients with neurological involvement could have future sequelae; however, long-term follow-up of patients with neurological involvement related to COVID-19 is necessary.

## Figures and Tables

**Table 1:** Distribution according to the age and sex of pediatric patients with COVID-19.

	NM Group n = 23	Non-NM Group n = 23	Total n = 46
	Mean (SD)		*p*-value
Age (years)	9.22 (4.56)	8.74 (4.8)	0.664
Sex			
Male n (%)	15 (65.2)	11 (47.8)	26 (56.5)
Female n (%)	8 (34.8)	12 (52.2)	20 (43.5)
Age range (years)			
<3 n (%)	1 (4.3)	3 (13.04)	4 (8.7)
3 to 5 n (%)	3 (13.04)	4 (17.4)	7 (15.2)
6 to 12 n (%)	13 (56.5)	7 (30.4)	20 (43.5)
12 to <18 n (%)	6 (26.1)	9 (39.1)	15 (32.6)
Total	23 (50)	23 (50)	46 (100)

Data are shown as frequencies and percentages. Abbreviations: NM, neurological manifestation; Non-NM, non-neurological manifestation.

**Table 2:** Distribution of the comorbidities in pediatric patients with COVID-19.

Comorbidity	NM Group n = 23	Non-NM Group n = 23	Total n = 46	*p*
ALL n (%)	13 (56.5)	8 (38.8)	21 (45.6)	0.091
AML n (%)	1 (4.3)	4 (17.4)	5 (10.9)	
Obesity n (%)	4 (17.4)	1 (4.3)	5 (10.9)	
Other n (%)	5 (21.7) *	10 (43.5) **	15 (32.6)	
Total n (%)	23 (100)	23 (100)	46 (100)	

Data are shown as frequencies and percentages. * Pinealoblastoma grade IV, bronchopulmonary dysplasia, alveolar rhabdomyosarcoma, mediastinal germ cell tumor, and pineal gland germinoma. ** Mature teratoma, right gonadal germ cell tumor, immature teratoma, myelodysplastic syndrome, Hodgkin’s lymphoma, medulloblastoma, hypohidrotic ectodermal dysplasia, chondroblastic osteosarcoma of the tibia, diabetes mellitus 1, and disease secondary to systemic lupus erythematosus. Abbreviations: NM, neurological manifestation; Non-NM, non-neurological manifestation; ALL, acute lymphoblastic leukemia; AML, acute myeloblastic leukemia.

**Table 3:** Clinical manifestations in pediatric patients with COVID-19.

Symptom	NM Group n = 23	Non-NM Group n = 23	Total n = 46
Fever n (%)	18 (78.2%)	13 (56.6)	31 (67.4)
General discomfort n (%)	9 (39.1)	4 (17.4)	13 (28.3)
Myalgia n (%)	1 (4.3)	4 (17.4)	5 (10.9)
Arthralgias n (%)	0 (0)	3 (13)	3 (6.5)
Chest pain n (%)	1 (4.3)	2 (8.7)	3 (6.5)
Cough n (%)	12 (52.2)	5 (21.7)	17 (37)
Dyspnea n (%)	10 (43.5)	3 (13)	13 (28.3)
Odynophagia n (%)	2 (8.7)	5 (21.7)	7 (15.2)
Rhinorrhea n (%)	3 (13)	0 (0)	3 (6.5)
Diarrhea n (%)	9 (39.1)	3 (13)	12 (26.1)
Abdominal pain n (%)	6 (26.1)	3 (13)	9 (19.6)
Headache n (%)	13 (56.6)	0 (0.0)	13 (28.3)
Encephalopathy n (%)	10 (43.5)	0 (0)	10 (21.7)
Epileptic seizures n (%)	4 (17.4)	0 (0)	4 (8.7)
Neuropathy n (%)	3 (13)	0 (0)	3 (6.5)
Ageusia n (%)	1 (4.3)	0 (0)	1 (2.2)
Anosmia n (%)	1 (4.3)	0 (0)	1 (2.2)
Encephalitis n (%)	1 (4.3)	0 (0)	1 (2.2)
Cerebral hemorrhage n (%)	1 (4.3)	0 (0)	1 (2.2)
Subarachnoid hemorrhage n (%)	1 (4.3)	0 (0)	1 (2.2)

Data are shown as frequencies and percentages.

**Table 4:** Results of laboratory tests and organ function in pediatric patients with COVID-19.

Variable	Mean (SD)		*p*-Value
	NM Group n = 23	Non-NM Group n = 23	
Hemoglobin (g/dL)	10.9 (2.9)	12.0 (1.9)	0.031
Hematocrit (%)	32.4 (7.2)	34.9 (5.2)	0.138
Leukocytes (miles/mm^3^)	4533.9 (4341.4)	5588.7 (4267.5)	0.361
Lymphocytes (miles/mm^3^)	946.1 (1071.9)	1366.5 (1059.5)	0.584
Platelets (miles/mm^3^)	193.1 (143.7)	191.6 (123.7)	0.274
Glucose (mg/dL)	88 (14.7)	87.0 (26.1)	0.824
Cr (mg/dL)	0.43 (0.15)	0.84 (2.28)	0.076
BUN (mg/dL)	16.3 (14.6)	13.04 (11.51)	0.775
AST (U/L)	52.8 (56.6)	71.6 (79.5)	0.394
ALT (U/L)	48.8 (41.4)	56.8 (43.4)	0.359
DHL (U/L)	651.5 (1207.1)	358.04 (336.01)	0.021
Sodium (mEq/L)	138.4 (2.9)	140.0 (2.9)	0.062
Potassium (mEq/L)	3.9 (0.45)	4.2 (0.50)	0.086
Calcium (mg/dL)	9.06 (0.41)	9.22 (0.84)	0.423
Ferritin (ng/mL)	2721.9. (5300.5)	1349.1 (1209.7)	0.042
Procalcitonin (ng/mL)	1.9 (4.5)	1.3 (4.7)	0.757
C-Reactive Protein (mg/L)	78.6 (130.4)	21.8 (41.1)	0.002
D-dimer (mg/L)	1.8 (2.6)	2.6 (4.5)	0.427
pH	7.39 (0.06)	7.39 (0.06)	0.320
Bicarbonate (mEq/L)	19.61 (5.31)	20.83 (3.57)	0.142
PCO2 (mmHg)	32.17 (10.95)	29.23 (7.77)	0.347
PO2 (mmHg)	70.03 (31.53)	64.23 (17.88)	0.028
Kirby quotient (PaO2/FiO2)	387.04 (338.91)	332.57 (169.28)	0.015

Data are shown as mean and standard deviation. Statistical analyses were performed by one-way, independent, *t*-test. Statistical significance: *p* < 0.05. Abbreviations: SD, standard deviation.

**Table 5:** Outcomes in pediatric patients with COVID-19.

Variable	Mean (SD)		OR (IC_95_%)	*p*-Value
	NM Group n = 23	Non-NM Group n = 23		
Asymptomatic discharge	16	22	0.10 (0.01 to 0.93)	0.02
Discharge with respiratory symptoms	2	1	2.10 (0.17 to 24.8)	0.50
Death	5	0	2.28 (1.612 3.22)	0.018

Data are shown as frequencies and percentages. Statistical analyses were performed using the Chi-squared test. Statistical significance: *p* < 0.05. Abbreviations: CI, confidence interval; NM, neurological manifestation; Non-NM, non-neurological manifestation.

## Data Availability

Datasets analyzed or generated during this study can be requested from the authors.
